# Explainable machine learning aggregates polygenic risk scores and electronic health records for Alzheimer’s disease prediction

**DOI:** 10.1038/s41598-023-27551-1

**Published:** 2023-01-09

**Authors:** Xiaoyi Raymond Gao, Marion Chiariglione, Ke Qin, Karen Nuytemans, Douglas W. Scharre, Yi-Ju Li, Eden R. Martin

**Affiliations:** 1 Department of Ophthalmology and Visual Sciences, The Ohio State University, Columbus, OH USA; 2 Department of Biomedical Informatics, The Ohio State University, Columbus, OH USA; 3 Division of Human Genetics, The Ohio State University, Columbus, OH USA; 4 Ohio State University Physicians Inc., Columbus, OH USA; 5 John P. Hussman Institute for Human Genomics, University of Miami, Miller School of Medicine, Miami, FL USA; 6 Dr. John T. MacDonald Foundation Department of Human Genetics, University of Miami, Miller School of Medicine, Miami, FL USA; 7 Department of Neurology, The Ohio State University Wexner Medical Center, Columbus, OH USA; 8 Department of Biostatistics and Bioinformatics, Duke University School of Medicine, Durham, NC USA; 9 Duke Molecular Physiology Institute, Durham, NC USA

**Keywords:** Machine learning, Alzheimer's disease

## Abstract

Alzheimer’s disease (AD) is the most common late-onset neurodegenerative disorder. Identifying individuals at increased risk of developing AD is important for early intervention. Using data from the Alzheimer Disease Genetics Consortium, we constructed polygenic risk scores (PRSs) for AD and age-at-onset (AAO) of AD for the UK Biobank participants. We then built machine learning (ML) models for predicting development of AD, and explored feature importance among PRSs, conventional risk factors, and ICD-10 codes from electronic health records, a total of > 11,000 features using the UK Biobank dataset. We used eXtreme Gradient Boosting (XGBoost) and SHapley Additive exPlanations (SHAP), which provided superior ML performance as well as aided ML model explanation. For participants age 40 and older, the area under the curve for AD was 0.88. For subjects of age 65 and older (late-onset AD), PRSs were the most important predictors. This is the first observation that PRSs constructed from the AD risk and AAO play more important roles than age in predicting AD. The ML model also identified important predictors from EHR, including urinary tract infection, syncope and collapse, chest pain, disorientation and hypercholesterolemia, for developing AD. Our ML model improved the accuracy of AD risk prediction by efficiently exploring numerous predictors and identified novel feature patterns.

## Introduction

Alzheimer’s disease (AD) is the most common late-onset neurodegenerative disorder, affecting nearly six million individuals in the United States^1^. Globally, more than 50 million people are living with AD and other dementias^2,3^. Late-onset AD typically affects individuals of age 65 and older^2,4^, but early symptoms or indicators including structural MRI can reveal changes as much as ten years before disease onset^5^. Other indicators like amyloid in cerebrospinal fluid or positron emission tomography scan show changes 15–20 years prior to AD cognitive symptoms^6,7^. AD poses a significant burden to patients’ families and society. In the final stage of the disease, patients need complete care. Unfortunately, there is no cure for AD at present; however, early detection is crucial^8^, allowing for early interventions and potentially improving treatment outcomes^9^.

Both genetic and non-genetic risk factors have been reported for AD. Age is the strongest risk factor for AD and the majority of AD patients show symptoms after 65 years of age, often considered the threshold for late-onset AD^2,4^. Other commonly cited risk factors for AD include low education, hypertension, diabetes, and smoking^2,3,10^. Genetic predisposition plays an important role in AD with the heritability estimates ranging from 58 to 79%^11^. The Apolipoprotein-E gene (*APOE)* is the most well-known genetic risk factor for AD^3,12^, but genome-wide association studies (GWASs) have identified more than 40 genetic loci to date for AD^2^. In recent years, polygenic risk scores (PRSs) have been proposed to aggregate genetic effects, from small to large, across the genome into a single measure of risk for each individual^13,14^. Typically, PRSs have been constructed to predict disease risk using weights that correspond to effects from case–control comparisons, such as logistic regression^15–17^. Some have considered weights based on models that use age at onset (AAO) information, such as survival analysis^18,19^. We suggest that weights for PRS may also be based on effect estimates from case-only linear regression models of AAO, and previous theoretical work^20^ has shown that such case-only AAO statistics have the potential to capture different information from case–control risk statistics.

The availability of International Classification of Diseases Tenth Revision (ICD-10) codes from electronic health records (EHRs) opens the door to evaluation of many more potential risk factors for developing AD. However, the high-dimension of ICD-10 codes in EHRs poses a challenge for traditional statistical models, such as logistic regression or Cox proportional hazards model. Machine learning (ML) methods provide an attractive and effective alternative to traditional statistical regression models, especially in situations where one has a large number of features/predictors. For example, XGBoost^21^ is a well-known ML package that works under the gradient boosting framework and has been shown to perform well in numerous prediction projects and ML competitions. Furthermore, SHapley Additive exPlanations (SHAP)^22^ can be used to visualize XGBoost results and show the relative contributions of different features to the model. The combination of XGBoost and SHAP can be used as an *explainable* ML model^22^, which maintains the accuracy of ML models while providing the distribution of the effects with direction for each variable to enhance the interpretability of the results.

Data from large-scale AD consortia, such as the Alzheimer Disease Genetics Consortium (ADGC) (n ~ 20,000), have increased the power for genome-wide screening of genetic variants and provide robust estimates of genetic effects for AD and related phenotypes. Together with the rich EHR and genetic data in the large-scale bio-repositories, such as UK Biobank (UKB) dataset (n ~ 500,000), we have available resources to mine the high-dimensional data using ML methods for identifying novel risk factors (both genetic and non-genetic) for AD. In this study, we built explainable ML models for the risk of developing AD and explored feature importance of genetic, non-genetic predictors and EHR ICD-10 codes using the ADGC and UKB datasets.

## Results

### Overview

**Figure 1:**
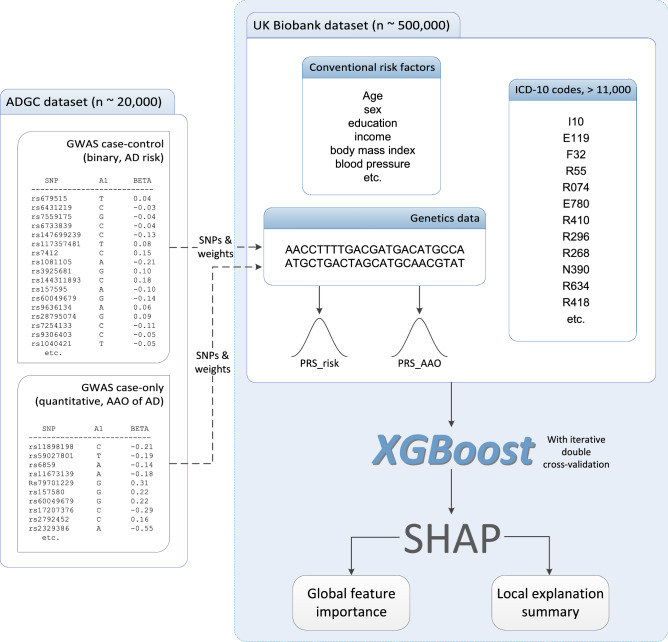
Flow chart of the study design. We evaluated prediction of development of Alzheimer’s disease (AD) in the UK Biobank (UKB) dataset using a large number of features/predictors, including polygenic risk scores (PRSs), conventional risk factors, and electronic health record ICD-10 codes, and state-of-the-art machine learning models. From the ADGC dataset, we conducted two GWASs, one for AD risk and the other one for age-at-onset of AD. We extracted significant genetic markers and their effect sizes (weights) from the GWAS summary statistics and applied them to the UKB dataset to derive two PRSs (PRS_risk_ and PRS_AAO_), for each individual in the UKB dataset. We then used the XGBoost (with iterative double cross-validation) and SHAP methods to build explainable machine learning models for the risk of developing AD and explored feature importance in the UKB dataset using the PRSs, conventional risk factors, and ICD-10 codes. *AAO* age at onset, *AD* Alzheimer’s disease, *ADGC* Alzheimer’s Disease Genetics Consortium, *GWAS* genome-wide association study, *PRS* polygenic risk score, *ICD-10* International Classification of Diseases Tenth Revision, *SHAP* SHapley Additive exPlanations, *SNP* single nucleotide polymorphisms.

A flowchart of our study design is shown in Fig. 1. We derived two sets of genome-wide association study (GWAS) summary statistics, treating Alzheimer’s disease (AD) as a binary phenotype and age-at-onset (AAO) of AD as a quantitative trait, using Alzheimer Disease Genetics Consortium (ADGC) datasets (supplementary Table 1). We calculated two polygenic risk scores (PRSs) from the binary and the quantitative ADGC GWAS summary statistics (statistical association testing results between genetic markers and a trait) for UK Biobank (UKB) participants (PRS_risk_ and PRS_AAO_). From a large number of features/predictors, including PRSs, conventional risk factors, and electronic health record (EHR) ICD-10 codes, we then evaluated prediction of development of AD in the UKB dataset using state-of-the-art machine learning (ML) models (from XGBoost) and cross-validation (CV).

### Non-genetic risk-factor characteristics in UKB

A total of 457,936 white participants from the UKB were included in our data analyses. Tables 1 and 2 show the characteristics for age 40 and older (age 40 + , n = 457,936) and age 65 and older (age 65 + , n = 88,309) groups. Among the age 40 + and age 65 + participants, 2177 and 1318, respectively, developed AD during the follow-up visits. The mean (SD) age at baseline of the eventual AD and non-AD subjects were 65.1 (4.3) and 57.2 (8.0) years, respectively. The mean systolic blood pressure (SBP) was higher and diastolic blood pressure (DBP) lower in AD cases than non-AD individuals and diabetes was more prevalent in cases. In addition, household, income, and education were lower, and falls in the past year, hearing difficulty, and mother’s AD history were higher in AD versus non-AD individuals. Among the age 65 + participants, similar patterns were seen except that the proportion of females and mean SBP appeared similar between AD and non-AD subjects.

**Table 1:** Comparison of subject characteristics between AD cases and non-AD controls (age 40 +).

Characteristics	Number of AD cases (n = 2177)	Number of non-AD controls (n = 455,759)
Age (years)	65.1 (4.3)	57.2 (8.0)
**Sex**		
Female	1129 (51.9%)	247,618 (54.3%)
Male	1048 (48.1%)	208,141 (45.7%)
Body mass index	27.4 (4.8)	27.4 (4.8)
Systolic blood pressure (mmHg)	144.6 (19.2)	137.9 (18.6)
Diastolic blood pressure (mmHg)	81.5 (10.2)	82.2 (10.1)
**Diabetes diagnosed by doctor**		
No	1918 (88.5%)	433,004 (95.2%)
Yes	249 (11.5%)	21,713 (4.8%)
Townsend deprivation index	− 1.2 (3.2)	− 1.5 (3.0)
**Household income (GBP/yr)**		
< 18,000	760 (47.0%)	86,681 (22.1%)
18,000 to 30,999	517 (31.9%)	99,799 (25.4%)
31,000 to 51,999	221 (13.7%)	103,422 (26.3%)
52,000 to 100,000	94 (5.8%)	81,104 (20.7%)
> 100,000	26 (1.6%)	21,522 (5.5%)
**Education**		
College/university	414 (19.5%)	146,142 (32.4%)
Non-college qualifications	928 (43.6%)	227,157 (50.3%)
None of the above	784 (36.9%)	78,031 (17.3%)
**Falls in the past year**		
No fall	1529 (70.7%)	365,517 (80.4%)
One fall	371 (17.2%)	59,896 (13.2%)
More than one fall	262 (12.1%)	29,479 (6.5%)
**Hearing difficulty with background noise**		
No	1067 (50.8%)	278,270 (62.3%)
Yes	1033 (49.2%)	168,571 (37.7%)
**Mother Alzheimer’s Disease history**		
No	1778 (83.5%)	416,677 (93.0%)
Yes	352 (16.5%)	31,595 (7.0%)

For continuous data, mean and standard deviation (sd) for each group were shown. Missing data percentage: Body mass index, 0.32%; Systolic blood pressure, 5.8%; Diastolic blood pressure, 5.8%; Diabetes diagnosed by doctor, 0.23%; Townsend deprivation index, 0.11%; Household income, 13.9%; Education, 0.98%; Falls in past year, 0.19%; Hearing difficulty with background noise, 2.0%; Mother Alzheimer’s Disease history, 1.6%.

**Table 2:** Comparison of subject characteristics between AD cases and non-AD controls (age 65 +).

Characteristics	Number of AD cases (n = 1318)	Number of non-AD controls (n = 86,991)
Age (years)	67.8 (1.5)	67.4 (1.5)
**Sex**		
Female	681 (51.7%)	44,086 (50.7%)
Male	637 (48.3%)	42,905 (49.3%)
Body mass index	27.4 (4.7)	27.6 (4.4)
Systolic blood pressure (mmHg)	146.3 (19.2)	145.8 (18.9)
Diastolic blood pressure (mmHg)	81.0 (10.2)	81.9 (10.0)
**Diabetes diagnosed by doctor**		
No	1147 (87.6%)	80,077 (92.3%)
Yes	163 (12.4%)	6669 (7.7%)
Townsend deprivation index	− 1.3 (3.1)	− 1.6 (2.9)
**Household income (GBP/yr)**		
< 18,000	499 (52.3%)	28,016 (40.5%)
18,000 to 30,999	308 (32.2%)	23,714 (34.3%)
31,000 to 51,999	106 (11.1%)	11,890 (17.2%)
52,000 to 100,000	35 (3.7%)	4530 (6.5%)
> 100,000	7 (0.7%)	1027 (1.5%)
**Education**		
College/university	214 (16.7%)	19,709 (23.0%)
Non-college qualifications	537 (41.8%)	38,174 (44.6%)
None of the above	533 (41.5%)	27,777 (32.4%)
**Falls in the past year**		
No fall	925 (70.8%)	67,041 (77.2%)
One fall	231 (17.7%)	13,692 (15.8%)
More than one fall	151 (11.5%)	6104 (7.0%)
**Hearing difficulty with background noise**		
No	625 (49.0%)	46,030 (54.0%)
Yes	650 (51.0%)	39,185 (46.0%)
**Mother Alzheimer’s Disease history**		
No	1083 (83.7%)	77,350 (90.4%)
Yes	211 (16.3%)	8188 (9.6%)

For continuous data, mean and standard deviation (sd) for each group were shown. Missing data percentage: Body mass index, 0.40%; Systolic blood pressure, 5.6%; Diastolic blood pressure, 5.6%; Diabetes diagnosed by doctor, 0.29%; Townsend deprivation index, 0.07%; Household income, 20.6%; Education, 1.5%; Falls in past year, 0.19%; Hearing difficulty with background noise, 2.0%; Mother Alzheimer’s Disease history, 1.7%.

### SHAP feature importance

**Figure 2:**
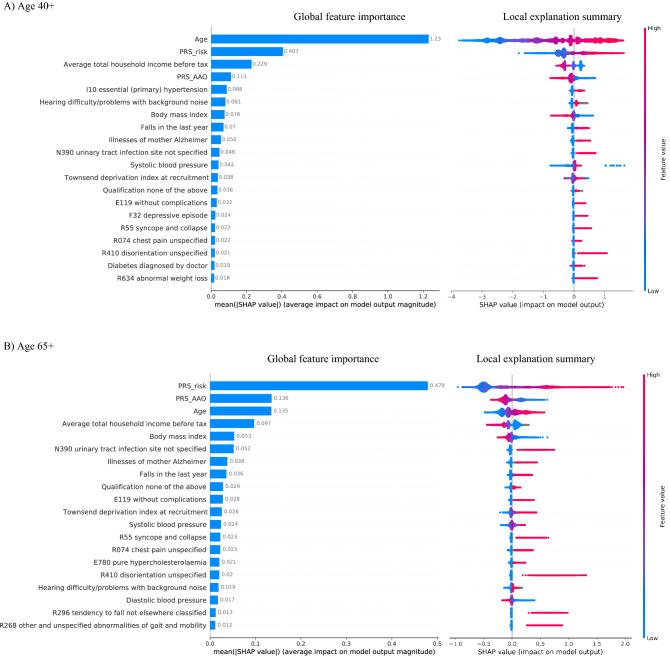
Feature importance and impact of top 20 most important features for different age groups. SHAP global feature importance bar chart (left) and SHAP local explanation summary plot (right) for two age groups (**A**) Age 40 + , and (**B**) Age 65 + . Red and blue in the summary plot represent high and low feature values, respectively. The gray numerical values in the left bar plots show the mean (|SHAP value|), which shows the average impact on model output magnitude. The long right tails in the local explanation summary plot indicate rare but large-effect-size risk factors.

Feature importance for XGBoost models was evaluated using SHAP values. Figure 2A and B show the top 20 features for age 40 + and age 65 + groups, respectively. The left panels show the bar plots of the global feature importance ranked from most important to least (top to bottom). For the age 40 + group (Fig. 2A bar plot), age ranks first and is the most important predictor. PRS_risk_ and PRS_AAO_ rank the second and the fourth, respectively. Other features that appear in the top 20 list include conventional risk factors (in SHAP feature importance sequential order), i.e. average household income, hearing difficulty problems with background noise, body mass index (BMI), falls in the last year, illness of mother Alzheimer’s, SBP, Townsend deprivation index (TDI) and education (qualification none of above, i.e. none of college/university or professional qualifications, detailed categories are shown in Supplementary Table 2), and diagnoses (ICD-10 codes), such as hypertension (I10), urinary tract infection (N390), diabetes (E119), depressive episode (F32), syncope and collapse (R55), chest pain unspecified (R074), disorientation (R410), and abnormal weight loss (R634). For the age 65 + group (Fig. 2B bar plot), the PRSs are the most important predictors, while age ranks the third in feature importance. The overall feature importance of PRS_risk_ is more than three times higher than that of age. Other features that appear in the top 20 list for age 65 + group include conventional risk factors (in SHAP feature importance sequential order), i.e. average household income, BMI, illness of mother Alzheimer’s, falls in the last year, education (qualification none of above), TDI, SBP, hearing difficulty problems with background noise, and DBP, and diagnoses, i.e. urinary tract infection (N390), diabetes (E119), syncope and collapse (R55), chest pain (R074), hypercholesterolaemia (E780), disorientation (R410), tendency to fall (R296), and abnormalities of gait and mobility (R268).

We also constructed local explanation plots, which summarize the overall distribution of SHAP values for all individuals and show the directions of the effects (right panels of Fig. 2A,B). From these plots, we can observe that higher PRS_risk_ pushes individuals to have higher odds for developing AD (higher SHAP values). Similar patterns are also seen for age, mother with AD, falls in the last year, lack of education (none of college/university or professional qualifications, detailed categories are shown in Supplementary Table 2), TDI, and comorbidities from EHR, such as diabetes, syncope and collapse, chest pain, disorientation, hypercholesterolaemia. The long right tails of several ICD-10 codes, such as N390 UTI, R410 disorientation, and R296 tendency to fall, indicate rare risk factors with large effect sizes. We also observed that PRS_AAO_, income, and BMI showed negative relationship with AD. For blood pressure, high SBP and low DBP show increased odds for developing AD.

### Model performance for different subgroups and selected features

**Figure 3:**
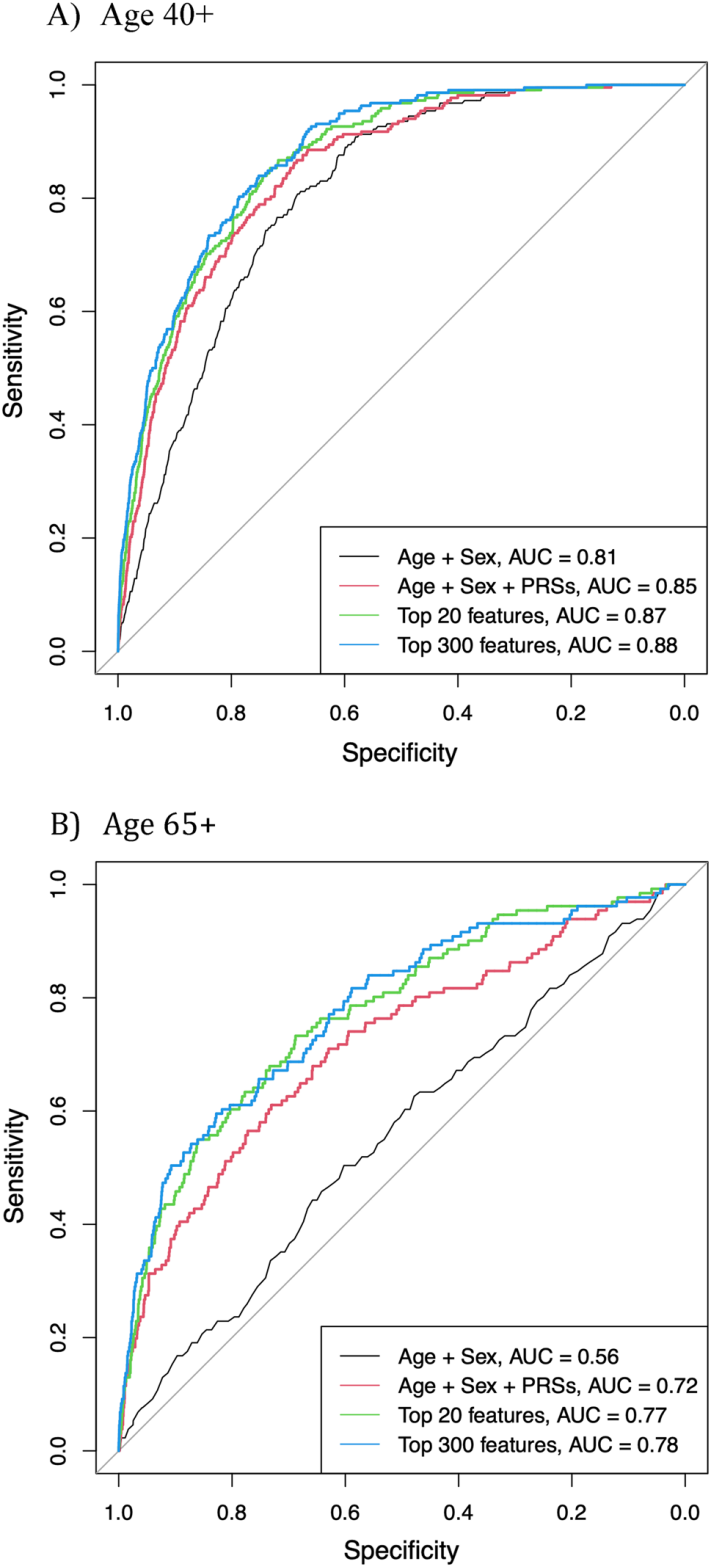
Receiver operating characteristic curves predicting Alzheimer’s disease. AUC curves for prediction accuracy of four models: (1) age and sex only, (2) age, sex, and PRSs, (3) the top 20 features, including PRSs, identified from XGBoost (features names shown in Fig. 3), (4) the top 300 features identified from XGBoost. (**A**) Age 40 + (age 40 and older) group; (**B**) Age 65 + (age 65 and older) group. A larger AUC representing better classification of Alzheimer’s disease status. *AUC* area under the receiver operating characteristic curve, *PRS* polygenic risk score.

We used XGBoost and tenfold cross-validation (CV) to examine the discriminatory ability of PRSs (PRS_risk_ and PRS_AAO_), conventional risk factors and EHR information captured in ICD-10 codes. Figure 3 displays the AUC results from a single, representative tenfold CV run using different prediction models in the two age groups: (A) 40 + and (B) 65 + . To evaluate the relative performance of including additional predictors, we considered four models: (1) age and sex only; (2) age, sex and the PRSs from the ADGC GWASs; (3) the top 20 features from XGboost, including PRSs (feature names shown in Fig. 2A,B); (4) the top 300 features from XGboost, including PRSs. For the age 40 + group, the AUC for the first model with only age and sex, was 0.81 (95% CI 0.77–0.84). Adding PRSs to the model yielded a significant increase (*p* = 2.61 × 10^–6^) in the mean AUC to 0.85 (95% CI 0.82–0.88). When the other non-genetic and ICD-10 features were added to the model, the estimated mean AUC increased to 0.87 for the top 20 features and 0.88 for the top 300 features. For the age 65 + group, age and sex only yield an AUC of 0.56 (95% CI 0.50–0.61). Adding PRSs to the model, the AUC has a significant 16% increase to 0.72 (95% CI 0.67–0.78; *p* = 8.03 × 10^–8^). Further adding other features, the model AUC reaches 0.77 and 0.78 for the top 20 and top 300 features, respectively. Box plots of the AUC results from the tenfold CV is shown in Supplementary Fig. S1.

### Logistic regression analysis

We further evaluated the risk factors identified by XGboost using traditional logistic regression analysis. Tables 3 and 4 display the odds ratio (OR) and p-value of these risk factors for single-feature logistic regression with adjustment for age and sex. In both age 40 + and age 65 + groups, most of top features show highly significant associations with AD except for BMI and SBP. The PRSs show highly significant associations with AD in both age 40 + and age 65 + groups. Results from the logistic regression analysis show consistent direction of effects as that observed in the SHAP plots.

**Table 3:** Logistic regression for the top 20 features from XGBoost (age 40 +).

Feature	OR	95% CI	*P*
Age	1.23	(1.218, 1.243)	3.13 × 10^−345^
PRS_risk_	3.215	(3.021, 3.421)	1.01 × 10^−295^
Average total household income before tax	0.726	(0.687, 0.767)	3.33 × 10^−30^
PRS_AAO_	0.43	(0.409, 0.453)	3.32 × 10^−232^
I10 essential primary hypertension	1.68	(1.536, 1.839)	1.32 × 10^−29^
Hearing difficulty problems with background noise	1.25	(1.142, 1.367)	1.09 × 10^−06^
Body mass index	0.992	(0.982, 1.002)	0.1112
Falls in the last year	1.355	(1.271, 1.445)	1.33 × 10^−20^
Illnesses of mother Alzheimer	1.873	(1.661, 2.112)	1.20 × 10^−24^
N390 urinary tract infection site not specified	3.269	(2.858, 3.739)	8.57 × 10^−67^
Systolic blood pressure	1.002	(1.000, 1.004)	0.0826
Townsend deprivation index at recruitment	1.046	(1.032, 1.061)	2.44 × 10^−10^
Qualification none of the above^†^	1.521	(1.387, 1.668)	5.40 × 10^−19^
E119 diabetes without complications	2.146	(1.883, 2.445)	2.36 × 10^−30^
F32 depressive episode	3.364	(2.858, 3.959)	2.87 × 10^−48^
R55 syncope and collapse	2.787	(2.369, 3.279)	4.61 × 10^−35^
R074 chest pain unspecified	1.991	(1.745, 2.270)	9.51 × 10^−25^
R410 disorientation unspecified	6.547	(5.323, 8.051)	6.83 × 10^−71^
Diabetes diagnosed by doctor	1.785	(1.555, 2.048)	1.57 × 10^−16^
R634 abnormal weight loss	3.064	(2.519, 3.727)	3.69 × 10^−29^

Single-feature logistic regression with adjustment for age and sex.

*OR* odds ratio.

^†^None of the following: college or university, A levels/AS levels, O levels/GCSEs, CSEs, NVQ or HND or HNC, other professional qualifications, e.g. nursing and teaching.

**Table 4:** Logistic regression for the top 20 features from XGBoost (age 65 +).

Feature	OR	95% CI	*P*
PRS_risk_	3.323	(3.065, 3.603)	5.24 × 10^−186^
PRS_AAO_	0.418	(0.392, 0.447)	2.03 × 10^−149^
Age	1.195	(1.151, 1.241)	2.23 × 10^−20^
Average total household income before tax	0.742	(0.687, 0.802)	4.74 × 10^−14^
Body mass index	0.991	(0.978, 1.004)	0.1668
N390 urinary tract infection site not specified	3.167	(2.685, 3.736)	1.40 × 10^−42^
Illnesses of mother Alzheimer	1.835	(1.574, 2.140)	9.56 × 10^−15^
Falls in the last year	1.304	(1.2, 1.417)	4.00 × 10^−10^
Qualification none of the above^†^	1.463	(1.305, 1.641)	7.87 × 10^−11^
E119 diabetes without complications	2.034	(1.731, 2.389)	5.54 × 10^−18^
Townsend deprivation index at recruitment	1.042	(1.024, 1.062)	6.85 × 10^−06^
Systolic blood pressure	1.001	(0.998, 1.004)	0.5491
R55 syncope and collapse	2.554	(2.083, 3.132)	2.05 × 10^−19^
R074 chest pain unspecified	2.048	(1.741, 2.409)	5.12 × 10^−18^
E780 pure hypercholesterolaemia	1.732	(1.517, 1.977)	4.91 × 10^−16^
R410 disorientation unspecified	6.471	(5.056, 8.282)	8.95 × 10^−50^
Hearing difficulty problems with background noise	1.225	(1.092, 1.375)	0.0005
Diastolic blood pressure	0.992	(0.987, 0.998)	0.0108
R296 tendency to fall not elsewhere classified	7.418	(5.585, 9.852)	1.47 × 10^−43^
R268 other and unspecified abnormalities of gait and mobility	6.429	(4.775, 8.655)	1.38 × 10^−34^

Single-feature logistic regression with adjustment for age and sex.

*OR* odds ratio.

^†^None of the following: college or university, A levels/AS levels, O levels/GCSEs, CSEs, NVQ or HND or HNC, other professional qualifications, e.g. nursing and teaching.

## Discussion

In the present study, we constructed PRSs for AD risk and AAO, built ML models for predicting the risk of developing AD, and explored feature importance among PRSs, conventional risk factors, and ICD-10 codes from EHRs. Our results showed that PRSs from risk and AAO tests both substantially improved the discriminatory ability for AD, especially for the age 65 + group, where adding PRSs increased AUC by 16% over the model with only age and sex. Interestingly, PRSs ranked on the top, even higher than age, in feature importance for the age 65 + group. To improve interpretability of the ML technique, we computed SHAP values for feature ranking and visualization. To our knowledge, this is the first report to develop predictive models for AD using genetic, non-genetic information, and ICD-10 codes from EHR in a large-scale cohort study using a modern explainable ML framework.

Our ML model identified strong effects of both age and PRSs, but the relative contributions change over time. Age is generally accepted as the greatest risk factor for AD^3,4^. Our results are in general agreement with this, with age ranking first among all features for the age 40 + group, which likely explains the higher AUC performance compared to the age65 + group. Among individuals who are age 65 and older, the well-accepted age cutoff for late-onset AD, genetic effects captured by the PRSs become much more important than age, with a SHAP value three times higher (Fig. 2B). Although age and genetics are both widely-recognized risk factors for AD^2,3^, we are unaware of any previous reports on the relative contribution between age and PRSs. The significant contribution of PRSs to AD highlights the need to consider genetic information in assessing AD risk, particularly in older individuals. Our approach was to combine PRSs capturing both the risk and AAO effects, and we found that both contribute substantially to the ML predictive model.

Not unexpectedly, we found strong contributions of several conventional AD risk factors. Income played a particularly important role in our results, ranking just after age and PRSs in terms of feature importance. Income can be a deciding factor for our living environment, the kinds of food we eat, the education level, access to care and consequently may directly and indirectly affect many health conditions, including AD. Other known risk factors for AD, such as family history of AD/dementia, hearing difficulty problems, diabetes, and blood pressure were also identified as important factors in AD development in our ML models. Obesity is typically considered as a risk factor for AD^2,3^; however, reports for BMI have had mixed findings^23,24^. We observed that being underweight increased individuals’ AD risk, which is evident in the local SHAP explanation plots (Fig. 2). It is possible that weight loss is an early sign of AD, but our use of incident cases assures that the BMI measurements were taken prior to clinical diagnosis, meaning that this is not merely the result of later-stage dementia, making it a useful pre-clinical biomarker. It is generally accepted high blood pressure is a risk factor for AD, which is what we observed for SBP; however, we also observed the opposite for DBP, that lower DBP increases the odds of developing AD (Fig. 2 local explanation summary plots).

A key finding of our study was that information captured in ICD-10 codes from EHRs can provide important information for prediction of AD. Many of the ICD-10 code related variables that appeared among the top 20 features in both age groups, are included in ICD-10 Chapter 18, symptoms, signs and abnormal clinical and laboratory findings, not elsewhere classified, such as R55, R074, R410, R296, R268, and R634, which may indicate early signs of developing AD for certain individuals. Urinary tract infection (N390) was shown to be an important predictor for AD, especially for age 65 + individuals. This may indicate diminished hygiene in some pre-dementia individuals. Depressive episode (F32), syncope and collapse (R55), and many cerebrovascular-related risk factors, such as diabetes (E119), hypercholesterolemia (E780), and possibly chest pain (R074) appeared in the top-20-feature list. Diabetes and hypertension (I10) are well known risk factors for many diseases, including AD. Hypercholesterolemia is another common health problem, which can cause many complications, such as chest pain, heart attack and stroke. Cholesterol plays an important role in AD affecting amyloid, tau, and gliosis^25^. Disorientation (R410) symptoms are not uncommon in those who may later develop dementia and could suggest early cognitive changes. Tendency to fall (R296) and abnormalities of gait and mobility (R268) could indicate early executive and attentional impairments in some individuals. Supplementary Tables 3 and 4 show the rest of ICD-10 codes possibly involved in AD (SHAP value > 0.005). This is the first time these factors were investigated all together in a data-driven report for AD, which provides a holistic views of feature relationship and importance on AD.

In the situations of modeling high-dimensional data, modern ML methods like XGBoost and SHAP have many advantages over traditional regression models. In our investigation, we used more than 11,000 features/predictors. Some of them may have relatively high correlation (e.g. SBP and DBP, education and income) and have variable amounts of missing data. The large number of features and missing values pose challenges to traditional regression models, such as logistic regression and Cox proportional hazards model. In contrast, we used XGBoost, which is an efficient open-source implementation of the gradient boosted trees algorithm, has clear advantages in situations of a large number of features, missing values and high-order interactions. Though ML models may suffer from the criticism that they are a black box, coupling them with the SHAP algorithm, we can build explainable ML models that are both accurate and interpretable. ML models may be complex and less intuitive than traditional approaches; however, with SHAP values, the ML models can become explainable with clear global significance and local explanation interpretations^22^. Our XGBoost and SHAP model identified relevant risk factors for AD among tens of thousands of predictors, confirming the effectiveness of our ML model. From a practical perspective, we found little difference in AUC going from the top-20 to top-300 features, suggesting that once a model is built, we may only need information on relatively few key phenotypic measures to predict in any individual.

This study is not without limitations. Our investigation through an ML approach, although promising, should be considered the first attempt to unravel the complex relationship between genetic, conventional risk factors, and ICD-10 codes and the development of AD. Feature importance does not imply a causal relationship. Nevertheless, top-rank features did provide key risk factors and improved prediction accuracy. There are many ways to derive PRSs and there is no consensus about which PRS approach is the best^26^. We used a clumping and thresholding approach for calculating PRSs, which may not be optimal. Choosing different linkage disequilibrium and p-value cutoffs for PRSs may further improve accuracy^14^. Despite this, we observed that our PRSs ranked the first among all the risk factors (even higher than age) in individuals of age 65 and older. We were also limited by the data accessible to us. We included only white subjects since other racial subsets were considerably smaller; thus our results require further validation in diverse cohorts. The accuracy of ICD-10 codes can be hindered by billing and clinic workflows^27^. Despite this, ICD-10 codes remain an important source for research and have led to numerous discoveries^28^. The possible misclassification in ICD-10 would presumably bias our results towards the null. Hence, the potential prediction accuracy is likely to be higher if perfect data are available and used.

In conclusion, we not only identified key features for developing AD but also built advanced explainable ML models to address the general challenge of AD early detection. We constructed PRSs for AD and evaluated their discriminatory ability in predicting incident AD in combination with conventional risk factors and ICD-10 codes from EHRs. For exploring the large number of predictors (> 11,000), we used an explainable ML framework, XGBoost and SHAP, which provided superior ML performance as well as aided ML model explanation. Our results indicated that PRSs played the most important role in AD prediction in age 65 + group. We also identified physical health indicators as that captured in ICD-10 codes that contributed important roles in AD prediction. Our findings highlight the critical role of including PRSs in AD risk assessment in addition to including traditional risk factors and physical health indicators as that captured in ICD-10 codes in evaluating the risk for developing AD. We further made our ML models freely available (see data availability statement). We believe that the key features and the ML models have the potential to aid the early detection of AD.

## Methods

### Ethics statement

For the ADGC dataset, written informed consent was obtained from study participants or from a caregiver, legal guardian, or other proxy^29^. UKB was approved by the North West Multi-Center Research Ethics Committee. All participants provided written informed consent. We obtained fully de-identified data. Our study adheres to the tenets of the Declaration of Helsinki.

### ADGC dataset and summary statistics

We used twenty cohorts (Supplementary Table 1) of the ADGC dataset to derive GWAS summary statistics for AD risk and AAO of AD for use as PRS weights. Details of these cohorts have been reported previously^29,30^. In brief, both AD case–control status and AAO of AD cases were collected from the ADGC participants, as well as age at exam, sex, and DNA. Genotypes for the ADGC dataset were imputed previously using the Haplotype Reference Consortium (HRC) reference panel on the Michigan Imputation Server^31^. For summary statistics for AD risk, we analyzed AD cases and controls as a binary trait using the regenie software^32^ adjusting for age, sex and the first 10 principal components (PCs). For summary statistics for AAO, we used linear regression analysis in case only using the linear mixed-effects model as implemented in regenie^32^ adjusting for sex and the first 10 PCs. These GWAS summary statistics provide weights to derive PRSs for individuals in the UKB dataset.

### UKB dataset

The UKB is an ongoing, large prospective cohort study for public health. Details regarding this cohort have been described elsewhere^33,34^. Briefly, the UKB recruited over half a million adult participants (40 to 70 years of age at enrollment) living in the United Kingdom who were registered with the National Health Service at the study baseline (2006–2010). Medical information (self-report and EHRs), family history, lifestyle information, as well as DNA samples, were collected. For this study, we restricted our analysis to white participants.

Genotyping, imputation and quality control steps of the UKB genetic dataset have been described previously^35^. Briefly, the UKB data were genotyped using either the UK BiLEVE Axiom Array (807,411 markers; n = 49,950) or the UKB Axiom Array (825,927 markers; n = 438,427). The data were further imputed based on the 1000 Genomes Project, UK10K, and HRC reference panels. After quality control, 92,693,895 genetic markers and 487,442 samples were included in the data release. We excluded variants with low imputation quality (info score < 0.3) and minor allele frequency < 0.5%, resulting in approximately 11.9 million variants for downstream analysis^14,36,37^.

### Polygenic risk scores

Risk and AAO GWAS summary statistics from the ADGC dataset were used to derive two PRSs for each individual in the UKB dataset. First, we selected independent SNPs using PLINK^38,39^ LD-based clumping with *r*^*2*^ < 0.3 and *p* < 5 × 10^–8^. Details of the SNPs included in PRSs are shown in Supplementary Tables 5 and 6. We then constructed two PRSs applying the different GWAS results (risk and AAO) to the risk alleles derived from the imputed and quality controlled UKB data and calculated weighted PRSs using PLINK^40^. The two PRSs (PRS_risk_ and PRS_AAO_) aim to capture different aspects of genetic information, which include the risk of getting AD (binary case–control outcome) and the AAO of AD (quantitative-trait outcome), respectively.

### Risk factors and ICD-10 codes

In addition to PRSs capturing genetic information, we included both common AD risk factors and ICD-10 codes available in UKB EHR records as potential risk factors. Common risk factors included age, sex, body mass index (BMI), blood pressure (both systolic blood pressure [SBP] and diastolic blood pressure [DBP]), diabetes, education, as well as history of mother having AD, household income, Townsend deprivation index (TDI), falls in the last year, and hearing difficulty problems, from the baseline data as risk factors in the model. We aggregated > 11,000 ICD-10 codes from the baseline data to identify disease-related information that may serve as early predictors of AD. AD information was extracted from ICD-10 codes, both G30 and F00, based on UKB AD classification. We excluded other types of dementia from analysis (as AD or non-AD). We used incident AD within ten years from the baseline, which was defined as the onset of AD after the baseline data collection and before 2021.

### Explainable machine learning

We constructed XGBoost^21^ models that aggregate information from PRSs, baseline characteristics (non-genetic factors), and ICD-10 codes for predicting incident AD. We considered two age groups: participants of age 40 and older (age 40 +) and age 65 and older (age 65 +). All individuals in the UK Biobank dataset are 40 years of age and older. Age 65 is a well-accepted age cutoff for late onset AD^2,4^. Thus, we used these two age groups in this study. AD was treated as a binary outcome, i.e. AD and non-AD. We used the area under the receiver operating characteristic curve (AUC) and tenfold CV to quantify the predictive ability of the XGBoost models. In each CV fold, machine learning models were trained on 90% of the data, and the remaining 10% of data were held out for performance evaluation. For hyperparameter tuning, we used Bayesian Optimization, within which there was another tenfold CV through the XGBoost.cv() function. To address the imbalance of AD cases to non-AD controls in the UKB cohort, we used weighted XGBoost and assigned higher class weight for AD cases during model training. For evaluating feature importance, we used SHAP values^22^, which are based on a game theoretic approach to rank feature importance objectively and help explain the output of ML models. Pairwise comparisons of AUC between different models were performed using Delong’s test^41^. As a comparison, we also included logistic regression and obtained p-values for the identified predictors adjusting for age and sex. XGBoost, SHAP, and other downstream statistical analyses were performed using Python (v3.7.0) and R (v3.6.3).

## Supplementary Information

Supplementary Information.

## Data Availability

The data used in this study is available to researchers upon approval of an application to the UK Biobank (https://www.ukbiobank.ac.uk/researchers/) and a material transfer agreement. Our XGBoost models can be found at the following web address: https://u.osu.edu/gao.1671/ad-xgboost-models/.
