# Supplementary material for: Explainable machine learning aggregates polygenic risk scores and electronic health records for Alzheimer’s disease prediction
Source: Sci Rep. 2023 Jan 9;13:450. doi: 10.1038/s41598-023-27551-1 (PMC9829871; doi:10.1038/s41598-023-27551-1)
Supplement: Supplementary file 1 — Supplementary Information. [file 41598_2023_27551_MOESM1_ESM.docx]

**Explainable machine learning aggregates polygenic risk scores and electronic health records for Alzheimer’s disease prediction**

Supplementary Information

**Figure Legends**

**Supplementary Figure S1. Box plots of prediction accuracy using a 10-fold cross-validation.**

Box plots for prediction accuracy of four models: 1) age and sex only, 2) age, sex, and PRSs, 3) top 20 features identified from XGBoost (features names shown in Figure 3), 4) top 300 features identified from XGBoost.

AUC, area under the receiver operating characteristic curve.

**Supplementary Figure S1. Box plots of prediction accuracy using a 10-fold cross-validation.**

1. Age 40+

B) Age 65+

**Supplementary Table 1. Summary of the Alzheimer’s Disease Genetics Consortium (ADGC) dataset**

| **Cohort** | **Cases** | **Controls** | **Total** |
| --- | --- | --- | --- |
| act1 | 140 | 827 | 967 |
| adc1 | 479 | 459 | 938 |
| adc2 | 242 | 217 | 459 |
| adc3 | 266 | 748 | 1014 |
| adc4 | 102 | 321 | 423 |
| adc5 | 120 | 350 | 470 |
| adc6 | 74 | 267 | 341 |
| adc7 | 513 | 955 | 1468 |
| adni | 138 | 554 | 692 |
| gsk | 652 | 920 | 1572 |
| mtv | 95 | 212 | 307 |
| ohsu | 127 | 480 | 607 |
| pfizer | 103 | 1390 | 1493 |
| rosmap1 | 123 | 387 | 510 |
| tarc1 | 317 | 308 | 625 |
| tgen2 | 95 | 1416 | 1511 |
| uks | 594 | 1147 | 1741 |
| umvumssm | 658 | 1233 | 1891 |
| upitt | 1161 | 1058 | 2219 |
| washu1 | 318 | 352 | 670 |
| **Total** | 6317 | 13601 | 19918 |

**Supplementary Table 2. Education qualifications**

| College or University degree |
| --- |
| A levels/AS levels or equivalent |
| O levels/GCSEs or equivalent |
| CSEs or equivalent |
| NVQ or HND or HNC or equivalent |
| Other professional qualification eg: nursing, teaching |
| None of the above |
| Prefer not to answer |

**Supplementary Table 3. Feature importance of SHAP value >= 0.005 (Age 40+)**

| **Feature** | **SHAP importance** |
| --- | --- |
| Age | 1.230 |
| PRS_risk | 0.407 |
| Average total household income before tax | 0.229 |
| PRS_AAO | 0.113 |
| I10 essential (primary) hypertension | 0.088 |
| Hearing difficulty/problems with background noise | 0.081 |
| Body mass index | 0.076 |
| Falls in the last year | 0.070 |
| Illnesses of mother Alzheimer | 0.056 |
| N390 urinary tract infection site not specified | 0.048 |
| Systolic blood pressure | 0.042 |
| Townsend deprivation index at recruitment | 0.038 |
| Qualification none of the above | 0.036 |
| E119 without complications | 0.032 |
| F32 depressive episode | 0.024 |
| R55 syncope and collapse | 0.022 |
| R074 chest pain unspecified | 0.022 |
| R410 disorientation unspecified | 0.021 |
| Diabetes diagnosed by doctor | 0.019 |
| R634 abnormal weight loss | 0.018 |
| Hearing difficulty/problems | 0.016 |
| Diastolic blood pressure | 0.015 |
| G40 epilepsy | 0.015 |
| R418 other and unspecified symptoms and signs involving cognitive functions and awareness | 0.013 |
| G20 parkinson's disease | 0.011 |
| R413 other amnesia | 0.011 |
| F329 depressive episode unspecified | 0.011 |
| R296 tendency to fall not elsewhere classified | 0.010 |
| Sex | 0.010 |
| Z864 personal history of psychoactive substance abuse | 0.010 |
| Z511 chemotherapy session for neoplasm | 0.010 |
| G319 degenerative disease of nervous system unspecified | 0.009 |
| I209 angina pectoris unspecified | 0.008 |
| M179 gonarthrosis unspecified | 0.008 |
| M171 other primary gonarthrosis | 0.008 |
| Z867 personal history of diseases of the circulatory system | 0.008 |
| H919 hearing loss unspecified | 0.007 |
| M199 arthrosis unspecified | 0.007 |
| I848 unspecified haemorrhoids with other complications | 0.007 |
| I259 chronic ischaemic heart disease unspecified | 0.007 |
| W18 other fall on same level | 0.007 |
| F059 delirium unspecified | 0.007 |
| R268 other and unspecified abnormalities of gait and mobility | 0.006 |
| R91 abnormal findings on diagnostic imaging of lung | 0.006 |
| F419 anxiety disorder unspecified | 0.006 |
| R101 pain localised to upper abdomen | 0.006 |
| R568 other and unspecified convulsions | 0.005 |
| Z538 procedure not carried out for other reasons | 0.005 |
| R194 change in bowel habit | 0.005 |
| Z515 palliative care | 0.005 |
| Z866 personal history of diseases of the nervous system and sense organs | 0.005 |
| T814 infection following a procedure not elsewhere classified | 0.005 |
| Z854 personal history of malignant neoplasm of genital organs | 0.005 |
| Z911 personal history of noncompliance with medical treatment and regimen | 0.005 |
| C795 secondary malignant neoplasm of bone and bone marrow | 0.005 |

**Supplementary Table 4. Feature importance of SHAP value >= 0.005 (Age 65+)**

| **Feature** | **SHAP importance** |
| --- | --- |
| PRS_risk | 0.479 |
| PRS_AAO | 0.136 |
| Age | 0.135 |
| Average total household income before tax | 0.097 |
| Body mass index | 0.053 |
| N390 urinary tract infection site not specified | 0.052 |
| Illnesses of mother Alzheimer | 0.038 |
| Falls in the last year | 0.036 |
| Qualification none of the above | 0.029 |
| E119 without complications | 0.028 |
| Townsend deprivation index at recruitment | 0.026 |
| Systolic blood pressure | 0.024 |
| R55 syncope and collapse | 0.023 |
| R074 chest pain unspecified | 0.023 |
| E780 pure hypercholesterolaemia | 0.021 |
| R410 disorientation unspecified | 0.020 |
| Hearing difficulty/problems with background noise | 0.019 |
| Diastolic blood pressure | 0.017 |
| R296 tendency to fall not elsewhere classified | 0.013 |
| R268 other and unspecified abnormalities of gait and mobility | 0.012 |
| R104 other and unspecified abdominal pain | 0.011 |
| I10 essential (primary) hypertension | 0.011 |
| F059 delirium unspecified | 0.011 |
| Z515 palliative care | 0.011 |
| G20 Parkinson's disease | 0.010 |
| Qualification college or university degree | 0.009 |
| Diabetes diagnosed by doctor | 0.008 |
| Z867 personal history of diseases of the circulatory system | 0.008 |
| R634 abnormal weight loss | 0.007 |
| F329 depressive episode unspecified | 0.007 |
| R418 other and unspecified symptoms and signs involving cognitive functions and awareness | 0.007 |
| R32 unspecified urinary incontinence | 0.007 |
| D509 iron deficiency anaemia unspecified | 0.006 |
| R413 other amnesia | 0.005 |
| M796 pain in limb | 0.005 |
| K529 non-infective gastro-enteritis and colitis unspecified | 0.005 |
| G40 epilepsy | 0.005 |
| R194 change in bowel habit | 0.005 |

**Supplementary Table 5. Single nucleotide polymorphisms included in genetic risk scores (PRS_risk)**

| SNP | Chromosome | Position | A0 | A1 | A1FREQ | BETA |
| --- | --- | --- | --- | --- | --- | --- |
| rs679515 | 1 | 207750568 | C | T | 0.198 | 0.043 |
| rs6431219 | 2 | 127862133 | T | C | 0.580 | -0.031 |
| rs7559175 | 2 | 127883899 | A | G | 0.247 | -0.039 |
| rs6733839 | 2 | 127892810 | T | C | 0.596 | -0.041 |
| rs147699239 | 5 | 177222290 | T | C | 0.967 | -0.134 |
| rs9501224 | 6 | 32792910 | T | C | 0.958 | -0.096 |
| rs117357481 | 8 | 43518630 | A | T | 0.958 | 0.082 |
| rs11999092 | 9 | 90552360 | A | G | 0.973 | -0.109 |
| rs1582763 | 11 | 60021948 | A | G | 0.630 | 0.033 |
| rs142034848 | 19 | 45176557 | G | A | 0.981 | -0.162 |
| rs111740474 | 19 | 45223490 | A | G | 0.984 | -0.143 |
| rs74607435 | 19 | 45235700 | C | T | 0.952 | 0.081 |
| rs2927438 | 19 | 45242107 | G | A | 0.233 | 0.051 |
| rs12459810 | 19 | 45249661 | T | C | 0.692 | -0.053 |
| rs2965169 | 19 | 45251156 | C | A | 0.614 | 0.049 |
| rs80168591 | 19 | 45284627 | A | G | 0.986 | -0.229 |
| rs2967668 | 19 | 45302951 | G | A | 0.885 | 0.061 |
| rs140824606 | 19 | 45311084 | A | G | 0.971 | -0.122 |
| rs28399637 | 19 | 45324138 | A | G | 0.667 | -0.103 |
| rs147711004 | 19 | 45337918 | A | G | 0.949 | -0.227 |
| rs4239533 | 19 | 45342241 | G | A | 0.282 | -0.036 |
| rs111371860 | 19 | 45345787 | T | A | 0.947 | 0.090 |
| rs2972558 | 19 | 45356141 | T | C | 0.312 | -0.044 |
| rs41289514 | 19 | 45359667 | G | A | 0.988 | -0.196 |
| rs12462573 | 19 | 45359706 | A | G | 0.699 | -0.070 |
| rs138607350 | 19 | 45363820 | G | T | 0.988 | -0.207 |
| rs183427010 | 19 | 45366498 | A | G | 0.991 | -0.267 |
| rs73050293 | 19 | 45379746 | G | A | 0.874 | 0.058 |
| rs6859 | 19 | 45382034 | G | A | 0.464 | 0.061 |
| rs79701229 | 19 | 45384931 | A | G | 0.984 | -0.240 |
| rs283808 | 19 | 45387034 | C | A | 0.962 | 0.124 |
| rs1160984 | 19 | 45403924 | T | C | 0.945 | 0.070 |
| rs405697 | 19 | 45404691 | G | A | 0.240 | -0.069 |
| rs7259620 | 19 | 45407788 | A | G | 0.602 | 0.101 |
| rs449647 | 19 | 45408564 | T | A | 0.842 | 0.096 |
| rs429358 | 19 | 45411941 | C | T | 0.767 | -0.262 |
| rs7412 | 19 | 45412079 | T | C | 0.938 | 0.151 |
| rs1081105 | 19 | 45412955 | C | A | 0.959 | -0.212 |
| rs3925681 | 19 | 45421100 | A | G | 0.627 | 0.097 |
| rs144311893 | 19 | 45423944 | T | C | 0.984 | 0.179 |
| rs157595 | 19 | 45425460 | G | A | 0.357 | -0.095 |
| rs60049679 | 19 | 45429708 | C | G | 0.911 | -0.141 |
| rs9636134 | 19 | 45432505 | T | A | 0.648 | 0.061 |
| rs28795074 | 19 | 45436657 | A | G | 0.919 | 0.085 |
| rs114533385 | 19 | 45436753 | T | C | 0.984 | -0.153 |
| rs7254133 | 19 | 45438554 | T | C | 0.662 | -0.107 |
| rs4263041 | 19 | 45438643 | G | A | 0.731 | 0.052 |
| rs5167 | 19 | 45448465 | G | T | 0.631 | -0.034 |
| rs12981350 | 19 | 45522181 | A | G | 0.541 | 0.034 |
| rs74359223 | 19 | 45522289 | A | G | 0.984 | -0.201 |
| rs112380717 | 19 | 45523061 | C | G | 0.982 | -0.183 |
| rs4803791 | 19 | 45523583 | A | G | 0.756 | -0.044 |
| rs112481437 | 19 | 45549135 | A | G | 0.974 | -0.130 |
| rs193187396 | 19 | 45557302 | A | G | 0.989 | -0.182 |
| rs78620885 | 19 | 45591084 | T | C | 0.968 | -0.122 |
| rs7248421 | 19 | 45606125 | T | C | 0.902 | -0.066 |
| rs146723120 | 19 | 45648220 | T | C | 0.987 | -0.172 |
| rs620807 | 19 | 45706952 | G | A | 0.691 | -0.037 |
| rs346739 | 19 | 45714525 | T | C | 0.362 | -0.038 |
| rs8109764 | 19 | 45722743 | A | G | 0.960 | -0.100 |
| rs532816383 | 21 | 14620000 | T | C | 0.984 | 0.310 |
| rs73148780 | 21 | 15064337 | T | C | 0.764 | -0.126 |
| rs2792452 | 21 | 15119875 | T | C | 0.470 | -0.097 |
| rs2260627 | 21 | 15152522 | G | C | 0.704 | -0.101 |
| rs28516815 | 21 | 16920721 | A | G | 0.986 | 0.161 |
| rs8131112 | 21 | 44693064 | G | A | 0.943 | 0.105 |
| rs976531 | 21 | 44703295 | T | C | 0.924 | 0.086 |
| rs759235 | 22 | 17267006 | T | C | 0.375 | -0.032 |
| rs9608356 | 22 | 25415198 | G | C | 0.889 | -0.057 |
| rs9306403 | 22 | 25426113 | A | C | 0.762 | -0.048 |
| rs1040421 | 22 | 25436904 | C | T | 0.844 | -0.053 |

**Supplementary Table 6. Single nucleotide polymorphisms included in genetic risk scores (PRS_AAO)**

| SNP | Chromosome | | Position | A0 | A1 | A1FREQ | BETA |
| --- | --- | --- | --- | --- | --- | --- | --- |
| rs11898198 | 2 | 225293000 | | T | C | 0.955 | -0.214 |
| rs59027801 | 3 | 65820856 | | C | T | 0.951 | -0.192 |
| rs2077232 | 3 | 75682933 | | T | C | 0.556 | -0.185 |
| rs73840340 | 3 | 75716051 | | T | C | 0.879 | -0.320 |
| rs5000996 | 6 | 205920 | | T | G | 0.949 | -0.272 |
| rs188528155 | 6 | 9250179 | | A | G | 0.720 | 0.104 |
| rs9501224 | 6 | 32792910 | | T | C | 0.945 | 0.286 |
| rs6951922 | 7 | 72003711 | | G | A | 0.928 | -0.169 |
| rs62467827 | 7 | 91277271 | | T | C | 0.987 | -0.473 |
| rs2078772 | 8 | 12542292 | | G | A | 0.566 | 0.130 |
| rs7463812 | 8 | 43504021 | | G | A | 0.048 | 0.337 |
| rs117357481 | 8 | 43518630 | | A | T | 0.970 | -0.541 |
| rs13280603 | 8 | 119534455 | | A | G | 0.684 | 0.092 |
| rs301433 | 9 | 4580651 | | A | G | 0.524 | 0.090 |
| rs2927437 | 19 | 45241638 | | G | A | 0.226 | -0.097 |
| rs12459810 | 19 | 45249661 | | T | C | 0.668 | 0.105 |
| rs28399637 | 19 | 45324138 | | A | G | 0.623 | 0.146 |
| rs147711004 | 19 | 45337918 | | A | G | 0.928 | 0.327 |
| rs2927468 | 19 | 45357939 | | G | A | 0.404 | 0.112 |
| rs6859 | 19 | 45382034 | | G | A | 0.503 | -0.140 |
| rs11673139 | 19 | 45383037 | | T | A | 0.926 | -0.187 |
| rs79701229 | 19 | 45384931 | | A | G | 0.975 | 0.316 |
| rs157580 | 19 | 45395266 | | A | G | 0.295 | 0.225 |
| rs449647 | 19 | 45408564 | | T | A | 0.867 | -0.243 |
| rs429358 | 19 | 45411941 | | C | T | 0.652 | 0.427 |
| rs7412 | 19 | 45412079 | | T | C | 0.957 | -0.289 |
| rs1081105 | 19 | 45412955 | | C | A | 0.939 | 0.290 |
| rs3925681 | 19 | 45421100 | | A | G | 0.676 | -0.224 |
| rs60049679 | 19 | 45429708 | | C | G | 0.891 | 0.221 |
| rs7254133 | 19 | 45438554 | | T | C | 0.622 | 0.167 |
| rs17207376 | 19 | 55248072 | | A | C | 0.467 | -0.297 |
| rs2792452 | 21 | 15119875 | | T | C | 0.410 | 0.162 |
| rs2329386 | 21 | 44687679 | | G | A | 0.957 | -0.554 |
| rs976531 | 21 | 44703295 | | T | C | 0.940 | -0.383 |
